# Growth of *Bifidobacterium* species is inhibited by free fatty acids and bile salts but not by glycerides

**DOI:** 10.3934/microbiol.2022005

**Published:** 2022-03-10

**Authors:** Sergio Perez-Burillo, Sumudu Rajakaruna, Oleg Paliy

**Affiliations:** 1 Department of Biochemistry and Molecular Biology, Boonshoft School of Medicine, Wright State University, Dayton, Ohio, USA; 2 Instituto de Nutrición y Tecnología de los Alimentos, Centro de Investigación Biomédica (CIBM), Granada, Universidad de Granada, Spain

**Keywords:** *Bifidobacterium*, fats, bile salts, fatty acids, glycerides

## Abstract

High-fat diets have been associated with lower gut and fecal abundances of genus *Bifidobacterium*. Here, we investigated whether commonly consumed dietary free fatty acids have any detrimental effect on the growth of *B. adolescentis*, *B. bifidum*, and *B. longum*. We found that the presence of free fatty acids in the medium inhibits the growth of *Bifidobacterium* species to a varying degree, with capric (C10:0), oleic (C18:1), and linoleic (C18:2) acids displaying the largest effect. In comparison, free fatty acids did not affect the growth of *Escherichia coli*. When fats were added as a mixture of mono- and diacylglycerols, the inhibitory effect on *Bifidobacterium* growth was abolished.

Dietary fats comprise a major part of human diet. Nutrient recommendations call for fats accounting for 20–35% of the daily calories [1]. The actual consumption of fats in western countries is significantly higher, providing around 40% of caloric intake [2]. While dietary fats are efficiently absorbed in the small intestine [3], increased dietary intake of fats likely saturates its absorption capacity [4], and so substantial amounts of lipids reach the colon along with some bile salts. In the colon, these compounds interact with resident gut microbiota [5],[6]. Recently, using an *in vitro* Human Gut Simulator system, we showed that human gut microbiota can efficiently utilize dietary free fatty acids for growth, and we identified specific “lipidophilic” and “lipidophobic” members [7]. Surprisingly, *Bifidobacterium* members were found in unexpectedly low numbers in all samples, even in the balanced medium that contained abundant carbohydrate and protein nutrients in addition to free fatty acids. Abundance of this genus was also reported to be low in several studies of high-fat diets, both in humans [8],[9] and in animal models [10].

The goal of this study was to determine if fatty acids, commonly consumed as part of human diet, and/or bile salts could have an inhibitory effect on the growth of several *Bifidobacterium* species. The most abundant colonic species of free fatty acids [7] were selected for testing and included capric (C10:0), palmitic (C16:0), stearic (C18:0), oleic (C18:1), and linoleic (18:2) acids. Three *Bifidobacterium* species with well-established prevalence in human gut (*Bifidobacterium longum* subsp. *longum* DSM-20219*, Bifidobacterium bifidum* DSM-20456*, Bifidobacterium adolescentis L2-32*) were grown in either DSMZ Medium 58 (“*Bifidobacterium* medium”, BM) as recommended by the German Collection of Microorganisms and Cell Cultures, or in the balanced Western diet medium (WM), which we used previously to mimic food bolus contents reaching colon in subjects consuming a typical Western diet [7]. *Escherichia coli* K12 NCM3722 [11], which was previously found to tolerate well the presence of fats in the growth medium [12], was used as a control, and was cultured in the Western diet medium.

The experimental design was as follows: each *Bifidobacterium* strain was cultured in both BM and WM media with or without fatty acids and/or bile salts. Control medium contained no fatty acids or bile salts. Fatty acids were tested individually as well as in mixture in concentrations matching the contents of the Western diet medium, which in turn was developed to match bolus contents reaching the colon [7]. Medium composition is detailed in Table 1. A commercially available mixture of mono- and diacylglycerols (Modernist Pantry, LLC) was also used. All cultures were grown in Hungate tubes at 37 °C under fully anaerobic conditions. Atmosphere was 85% of N_2_, 10% of CO_2_, and 5% of H_2_. All Hungate tubes were inoculated to the same starting density from a single pre-culture tube. All experiments were performed in triplicates. After 24 hours of growth, cell densities of each culture were obtained via phase contrast microscopy with a Spencer hemocytometer as we did previously [13]. Statistical significance of culture density differences between samples was assessed with Student's t-test at a confidence level of 95%. Raw p-values were adjusted for multiple hypothesis testing according to Benjamini and Hochberg method [14].

**Table 1.** Medium composition, g l^−1^.

Medium component	BM	BM with added fats	WM control	WM
*Carbohydrates*				
Arabinogalactan	-	-	1.8	1.8
Guar gum	-	-	0.9	0.9
Inulin	-	-	0.9	0.9
Pectin	-	-	1.8	1.8
Starch	-	-	4.4	4.4
Xylan	-	-	0.9	0.9
Cellobiose	-	-	0.9	0.9
Glucose	10	10	0.5	0.5
Fructose	-	-	0.5	0.5
*Proteins*				
Peptone	10	10	3.3	3.3
Casein	-	-	2.0	2.0
*Lipids*				
Capric acid (C10:0)	-	0.3	-	0.3
Palmitic acid (C16:0)	-	1.5	-	1.5
Stearic acid (C18:0)	-	0.7	-	0.7
Oleic acid (C18:1)	-	1.8	-	1.8
Linoleic acid (C18:2)	-	1.2	-	1.2
*Mucin*	*-*	*-*	*4.0*	*4.0*
*Yeast extract*	*5.0*	*5.0*	*3.0*	*3.0*
*Meat extract*	*5.0*	*5.0*	*-*	*-*
*Soy extract*	*5.0*	*5.0*	*-*	*-*
*Salts, other components*	*22.5*	*22.5*	*14.9*	*14.9*
Bile salts	-	1.0	-	1.0

BM–*Bifidobacterium* medium; standard medium contained no fatty acids or bile salts.

WM–Western diet medium; control medium contained no fatty acids or bile salts.

All three tested *Bifidobacterium* species reached stationary phase after 24 hours of incubation. While there was some variability in the final cell densities reached, the values were consistent for each species across replicate cultures. All three species experienced inhibition by fatty acid pool (containing five different free fatty acids), and *B. bifidum* and *B. longum* were also significantly inhibited by bile salts (Figure 1). Overall, *B. longum* and *B. bifidum* were similarly affected across tested conditions. The presence of capric acid (C10:0), oleic acid (C18:1), and linoleic acid (18:2) had statistically significant negative effect on culture growth, whereas palmitic (C16:0) and stearic (C18:0) acids had no effect. On the other hand, *B. adolescentis* was not inhibited by bile salts but was significantly affected by each of the five tested free fatty acids. Oleic and linoleic acids have been previously observed to be inhibitory towards Gram-positive but not Gram-negative bacteria [12] including inhibition of *B. breve* [15]. Exposure to linoleic acid was found to alter *B. breve* metabolism triggering an increased oxidative stress that resulted in the lower growth rate [15]. Generally, free fatty acids possess membrane-destabilizing activity due to their amphipathic structure [16], and unsaturated fatty acids can also elicit bacteriostatic properties through the inhibition of fatty acid synthesis [12]. However, no inhibitory effect on *Bifidobacterium* was found in another investigation that tested the antimicrobial effect of different oils, all containing oleic and linoleic acids [17].

In our experiments, it was capric acid that showed the largest inhibitory effect across all three *Bifidobacterium* strains (Figure 1). To the best of our knowledge, capric acid's inhibitory effects on *Bifidobacterium* have not been tested previously, though it was shown to be inhibitory to other bacteria, possibly due to the disruption of ion transport through the membrane [18]. Interestingly, the concentration of capric acid in breast milk and breastfeeding formula was negatively correlated with the total relative abundance of *Bifidobacterium* in infants' fecal microbiota [19]. On the other hand, palmitic and stearic acid did not affect the growth of either *B. longum* or *B. bifidum,* which is consistent with other studies that found no harmful effects of saturated long-chain fatty acids on certain Gram-positive bacteria [12]. Surprisingly, our observations show that both of these fatty acids are able to hinder the growth of *B. adolescentis*, which had not been described previously.

**Figure 1.**
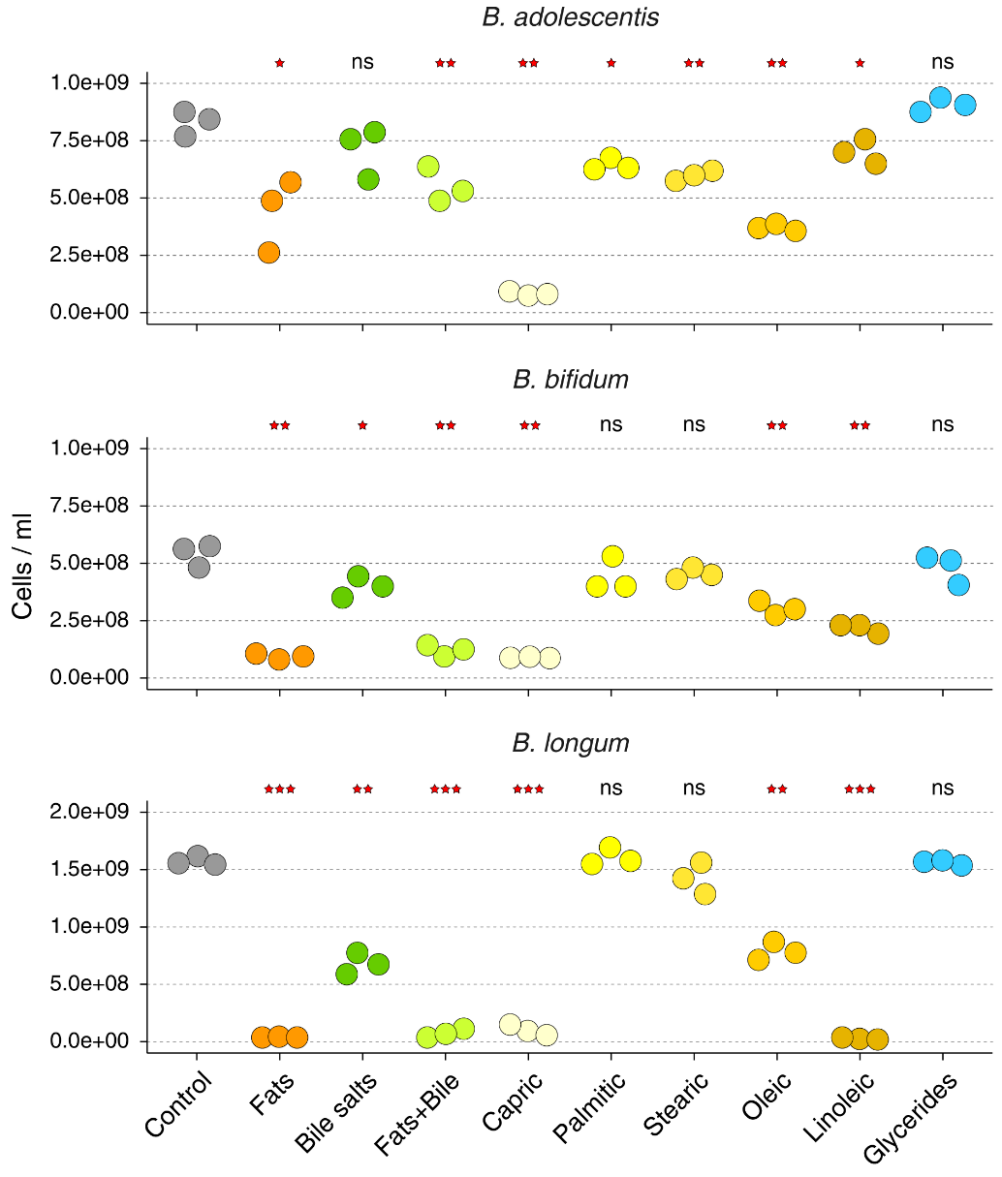
Cell densities of *Bifidobacterium* stationary phase cultures grown in *Bifidobacterium* medium. “Fats” denote a mixture of five tested fatty acids. Statistical significance was obtained via Student's one-tail t-test with α = 0.05 and multiple hypothesis testing adjustment. Statistical comparisons were made using the control as reference group. Significance labels: *: *q* < 0.05; **: *q* < 0.01; ***: *q* < 0.001; ns: not significant.

Bile salts are also known to possess antimicrobial properties due to their ability to alter cell membrane and to cause DNA damage [20], and the amount of bile salts released into the intestinal lumen correlates with the total fat content in consumed foods [21]. Thus, we also tested whether bile salts (a mixture of sodium glycocholate and sodium taurocholate) can have similar inhibitory effect on the *Bifidobacterium* growth. Tested *Bifidobacterium* species differed in their response to bile salts presence in the BM: bile had the largest inhibitory effect on the growth of *B. longum*, statistically significant inhibition of *B. bifidum*, and no statistically significant effect on the growth of *B. adolescentis*. *Bifidobacterium* strains that inhabit the gut may have developed various mechanisms to overcome the inhibition by bile acids. These adaptations include efflux of bile salts from cells, bile salt hydrolysis, and structural and compositional changes of bacterial cell membranes [20],[22]. All three tested *Bifidobacterium* species indeed encode bile salt hydrolase (EC:3.5.1.24, KEGG orthology: K01442) in their genomes.

In human gastrointestinal tract, majority of fatty acids reach the colon as partially hydrolyzed mono- or diacylglycerols. Therefore, we also tested an ability of a commercial mixture of mono and diacylglycerols (“glycerides”) to promote or inhibit *Bifidobacterium* growth. According to the manufacturer, this mixture can contain esterified caprylic (C8:0), capric (C10:0), lauric (C12:0), myristic (C14:0), palmitic (C16:0), stearic (C18:0), and oleic (C18:1) acids, with linoleic (C18:2) and linolenic (C18:3) acids being present in lesser amounts. This glyceride mixture did not exert any significant effect on any of the *Bifidobacterium* strains when they were cultured in BM. Because free hydroxyl groups of glycerol lower overall hydrophobicity of partially esterified acylglycerols and increase their polarity, this might lower their inhibitory effect on *Bifidobacterium* growth via decreased interactions with bacterial cell membranes.

Next, we carried out the same set of experiments using Western diet medium that has been developed to maintain complex human gut microbiota in the *in vitro* Human Gut Simulator system [7]. The results of this set of experiments generally matched those observed for the BM (Supplementary Figure S1). Specifically, all three *Bifidobacterium* species were significantly inhibited by capric, oleic, and linoleic acids. Bile salts were only inhibitory to *B. longum*. The mono- and diacylglycerol mixture had no detrimental effect. Comparing the three *Bifidobacterium* species, *B. bifidum* was the most resistant to growth inhibition across different testing conditions.

Because *Escherichia* and total Gammaproteobacteria were found to increase on fats-only medium in our recent study [7], we used this genus of Gram-negative bacteria as a resistant control. *E. coli* was cultured in WM under the same experimental setup. Free fatty acids and their mixtures had no significant inhibitory effect on *E. coli* growth (Table 2), which is congruent with our previous observations [7] and those of other studies [12]. The observed resistance can likely be attributed to the outer membrane of Gram-negative bacteria being a barrier to hydrophobic compounds [23].

**Table 2.** Cell densities of *E. coli* stationary phase cultures.

Medium composition	Cells ml^−1^
WM control	1.10 x 10^9^ ± 1.83 x 10^8^
WM + fatty acids and bile salts	1.07 x 10^9^ ± 7.64 x 10^7^
WM + fatty acids	0.97 x 10^9^ ± 5.91 x 10^7^
WM + bile salts	1.12 x 10^9^ ± 8.13 x 10^7^

WM-Western diet medium

Data are shown as arithmetic mean (n = 3) ± standard deviation

In conclusion, our results indicate that the presence of some free fatty acids in the environment, specifically capric, oleic, and linoleic acids, can hinder *Bifidobacterium* growth. Bile salts also inhibited *Bifidobacterium* growth but to a lesser degree. The mixture of mono- and diacylglycerols had no detrimental growth effect. We propose that when dietary fats are provided to gut microbiota community as part of growth medium, the free fatty acids are substituted with acylglycerols to mitigate any inhibitory effects on specific microbiota members.
