# Supplementary material for: Growth of Bifidobacterium species is inhibited by free fatty acids and bile salts but not by glycerides
Source: AIMS Microbiol. 2022 Mar 10;8(1):53–60. doi: 10.3934/microbiol.2022005 (PMC8995189; doi:10.3934/microbiol.2022005)
Supplement: Supplementary file 1 [file microbiol-08-01-005-s001.pdf]

AIMS Microbiology

AIMS Microbiology, 8(1): 53–60.
DOI: 10.3934/microbiol.2022005
Received: 01 December 2022
Revised: 25 February 2022
Accepted: 06 March 2022
Published: 10 March 2022

http://www.aimspress.com/journal/microbiology

Communication

Growth of *Bifidobacterium* species is inhibited by free fatty acids and bile salts but not by glycerides

Sergio Perez-Burillo[1,2], Sumudu Rajakaruna[1] and Oleg Paliy [1,*]

[1] Department of Biochemistry and Molecular Biology, Boonshoft School of Medicine, Wright State University, Dayton, Ohio, USA
[2] Instituto de Nutrición y Tecnología de los Alimentos, Centro de Investigación Biomédica (CIBM), Granada, Universidad de Granada, Spain

* **Correspondence:** Email: oleg.paliy@wright.edu.

Supplementary

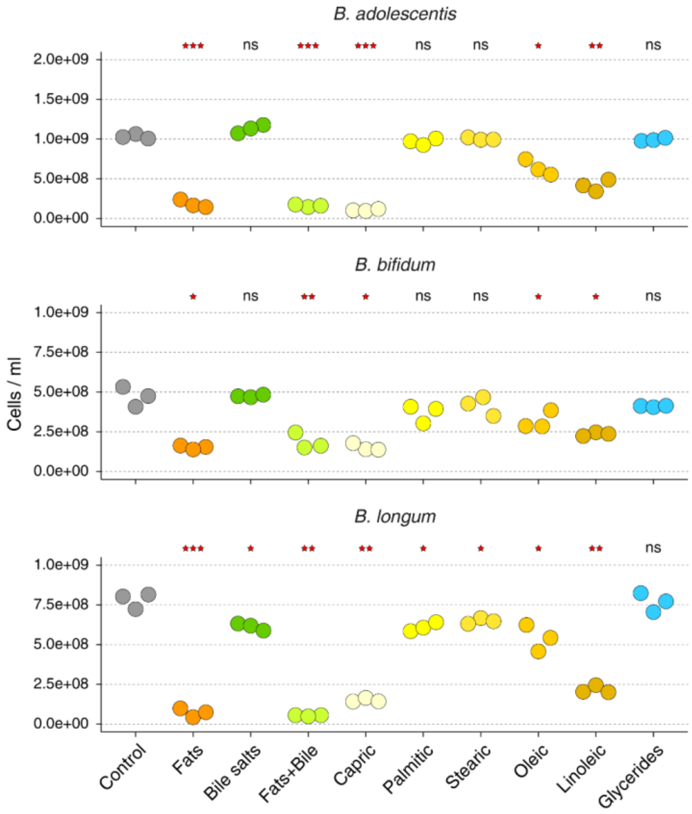

Supplementary Figure S1. Cell densities of *Bifidobacterium* stationary phase cultures grown in Western diet medium. "Fats" denote a mixture of five tested fatty acids. Statistical significance was obtained via Student's one-tail t-test with $\alpha = 0.05$ and multiple hypothesis testing adjustment. Statistical comparisons were made using the control as reference group. Significance labels: *: $q < 0.05$; **: $q < 0.01$; ***: $q < 0.001$; ns: not significant.

AIMS Press

© 2022 the Author(s), licensee AIMS Press. This is an open access article distributed under the terms of the Creative Commons Attribution License (http://creativecommons.org/licenses/by/4.0)
